# *Entropy* 2019 Best Paper Award

**DOI:** 10.3390/e22010124

**Published:** 2020-01-20

**Authors:** Entropy Editorial Office

**Affiliations:** MDPI, St. Alban-Anlage 66, 4052 Basel, Switzerland; entropy@mdpi.com

On behalf of the Editor-in-Chief, Prof. Dr. Kevin H. Knuth, we are pleased to announce the Entropy Best Paper Awards for 2019.

Papers published in 2018 were preselected by the *Entropy* Editorial Office based on the number of citations and downloads from the website. The winner nominations were made by a selection committee, which was chaired by the Editor-in-Chief and supported by thirteen Editorial Board Members. The three top-voted papers, in no particular order, have won the 2019 Entropy Best Paper Award:

**Levitated Nanoparticles for Microscopic Thermodynamics—A Review** 

**Jan Gieseler and James Millen** 

***Entropy* 2018, *20*(5), 326; https://doi.org/10.3390/e20050326** 

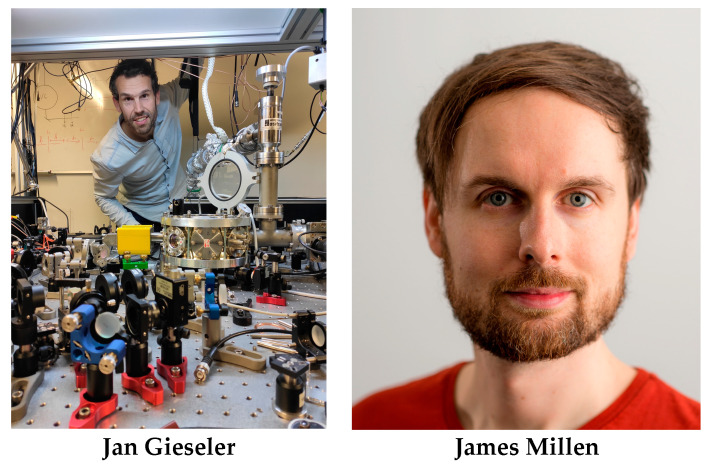

Technology is continuously miniaturizing, and as it reaches the nanoscale we face unique challenges. How do we control such small objects? What happens when temperature fluctuations have the same energy scale as our devices? When do quantum effects become important? In this review, we explore optical tweezers deployed in air (or partial vacuum) to levitate nanoscale objects, which creates an ideal system for exploring these questions.

Imagine a tiny sphere of glass. By tightly focusing a beam of light it can be trapped in a harmonic potential, a scenario known as optical tweezers. Arthur Ashkin was jointly awarded the Nobel Prize in Physics in 2018 for developing this technique. The motional energy of the sphere trapped in the optical tweezer is of the same scale as the thermal energy of the environment. This means that statistical fluctuations in the transfer of energy from the surroundings to the sphere are significant, and can be tracked, yet the fluctuations are not large enough to knock the particle out of the optical trap. This phenomenon allows one to study stochastic thermodynamics, which applies to small systems where fluctuations matter. For example, the second law of thermodynamics imposes strong constraints on the average work performed by the external forces and on the average heat dissipated towards the heat bath, but it does not impose constraints on the instantaneous fluctuations. The constraints on the mean values are certainly sufficient to characterize macroscopic systems, but not to characterize small systems, where fluctuations are non-negligible.

The optical forces acting upon the levitated sphere can be precisely controlled by altering the properties of the trapping laser beam. In this way, energy can be pumped into, or extracted from, the motion of the sphere. In addition, by moving from atmospheric conditions to vacuum, the motion of the sphere will change from being heavily damped to very underdamped. This control allows us to test a wide range of thermodynamic phenomena. These kinds of experiment are useful for a better understanding of the energetic efficiency and of the heat exchanges in nanosystems, for example, a Carnot cycle for nanomotors and the heat transfer between two coupled nanodevices at different temperatures, where the thermodynamic variables such as work, heat and entropy fluctuate. The study of the statistical properties of the thermodynamic variables is interesting because fluctuations may induce new and totally unexpected effects, such as heat flow from cold to hot or work extraction from a heat bath.

Recently, the first signatures of quantum mechanical motion have been observed in the dynamics of optically levitated nanoparticles. Hence, in the near future, the rich and paradigmatic system of an optically levitated nanoparticle may help us understand the thermodynamic interaction between the environment and a quantum particle.

**Transductive Feature Selection Using Clustering-Based Sample Entropy for Temperature Prediction in Weather Forecasting** 

**Zahra Karevan and Johan A.K. Suykens** 

***Entropy* 2018, *20*(4), 264; https://doi.org/10.3390/e20040264** 

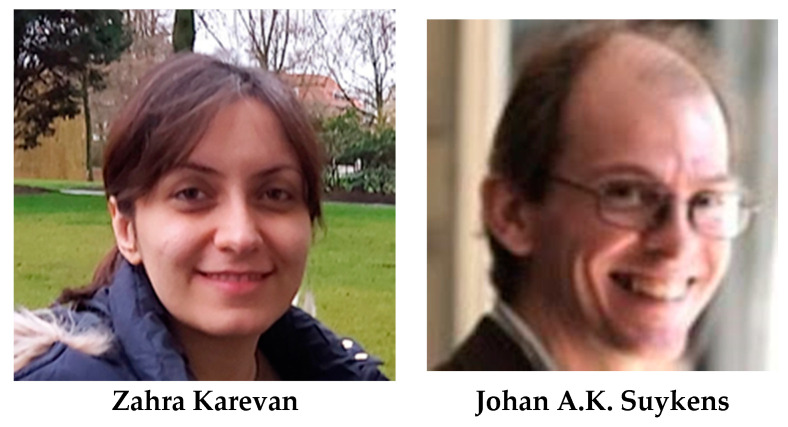

Entropy measures have been used to characterize the amount of information that a system contains. In the context of dynamical systems and time series, entropy measures can be utilized to illustrate the strength and the direction of causality. In this case, entropy measures the amount of information in one particular state of a process when the history of another is known. On the other hand, in time series analysis, the historical values of a variable are considered as input features of the function estimator. Hence, considering a longer history for the variable leads to a larger number of features. In complex systems, such as the weather system, where there are lots of variables involved in the interactions, taking into account the history of all variables results in a high dimensional problem. In such cases, feature selection can improve the performance and the interpretability of the model.

In our study, we compared a global and a transductive (local) feature selection approach based on an entropic measure for an application of weather forecasting. In the global approach, a global model is fitted on all data points; i.e., the same weights are considered for all data points in the training data for model fitting. In the transductive approach, the samples in the test point vicinity in feature space are considered to be more influential. In our study, we used sample entropy as an entropic measure. In order to find globally relevant features in forecasting, we deployed the lag-specific information transfer. In this approach, the information that each lag of a variable is giving to the current state of another variable is evaluated. In addition, by taking into account the local structure of the data, we proposed a clustering-based sample entropy methodology. In this approach, depending on the clustering information of the training data and the membership values of the test point to the clusters, the samples have different impact on the sample entropy. 

To evaluate the methods, we first performed the experiments on linear and nonlinear synthetic datasets. We generated 10 realizations of four dynamical systems, two of which (one linear and one nonlinear) have global behaviors; i.e., the relations between the variables are the same in all parts of the feature space. The other two have local behaviors; i.e., in different parts of the feature space the relations between the variables are different. Experimental results on the synthetic datasets indicate that in global systems, both global and transductive approaches are able to identify the relevant features. However, when the dynamical system is localized (has local behavior related to the function estimator), the transductive approach outperforms the global approach. In addition, we evaluated the performance of the global and transductive approaches on an application of temperature prediction in weather forecasting. The results in this case also indicate that the transductive approach outperforms the global approach in many cases. 

The proposed method is in principle applicable to many time-series prediction problems, such as climate, financial or medical systems, since it investigates the impact of the regressor of the time series on the target. Possible differences in patterns between summer and winter was a main intuition for using our model in weather forecasting. The transductive method is suitable for cases where the relations between variables in different parts of the input space are different. This can be the case in many other real-world applications, where the proposed method can be further explored in the future.

**Pointwise Partial Information Decomposition Using the Specificity and Ambiguity Lattices** 

**Conor Finn and Joseph T. Lizier** 

***Entropy* 2018, *20*(4), 297; https://doi.org/10.3390/e20040297 **

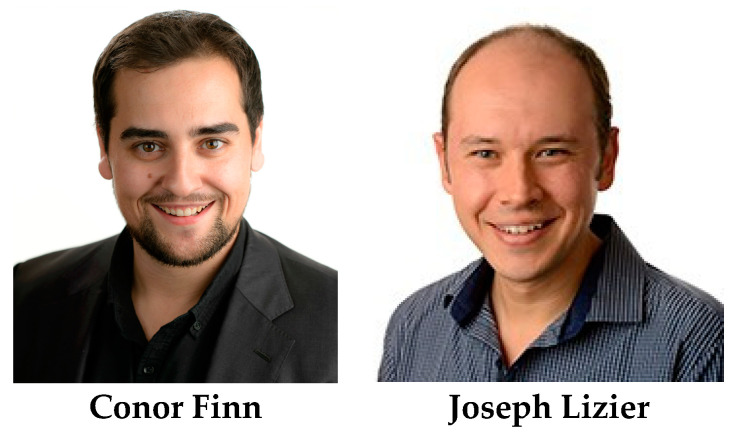

When studying real-world complex systems, such as the financial markets or genotype-phenotype mapping, we frequently seek to determine the strength of the interactions between the various components of the system. Given its ability to quantify both linear and non-linear interaction without assuming an underlying model, information theory is frequently able to quantify these dependencies. However, it has recently become clear that multivariate information measures can conflate different types of multi-component interdependence—that is, the unique, redundant and synergistic information. Since this issue was first highlighted nearly a decade ago, much theoretical progress has been made in information decomposition XF, i.e., the area of research that aims to develop methods for separately quantifying these distinct modes of dependency. Nevertheless, one of the key shortcomings with most of the existing approaches is their inability to provide a local or pointwise information decomposition.

Our study addresses this gap by providing a pointwise partial information decomposition. The key difficulties with pointwise information theory, is the fact that the pointwise mutual information can be negative. To overcome this difficulty, we apply the partial information decomposition separately to the unsigned entropic components of pointwise mutual information, which we refer to as the specificity and ambiguity. This yields a separate redundancy lattice for each component. We then define measures of redundant specificity and ambiguity using an operational definition based upon the idea of probability mass exclusions. Intuitively, the link between information and probability mass exclusions is exemplified by guessing games such Twenty Questions or Guess Who?—the more possibilities an inquiry excludes, the greater the amount information you received from the response to your query. Our operational definition of redundancy is then based upon the notion that the same information should make the same probability mass exclusions. Using these definitions of redundant specificity and ambiguity, we can then evaluate the partial information atoms in each lattice, which can be recombined to yield the sought-after pointwise partial information decomposition.

In the latter half of our study, we apply this framework to canonical examples from the literature and discuss the results and the various properties of the decomposition. Perhaps the most noteworthy of these properties is the fact that the pointwise decomposition, using specificity and ambiguity, satisfies a chain rule over target variables, which provides new insights into the so-called two-bit-copy example. We finish by discussing how these ideas are related to the notion of information provided by Kelly gambling.

**Prize Awarding Committee** 

Entropy Editorial Board.

